# Intervention with Microfinance for AIDS and Gender Equity (IMAGE): Women’s Engagement with the Scaled-up IMAGE Programme and Experience of Intimate Partner Violence in Rural South Africa

**DOI:** 10.1007/s11121-019-01070-w

**Published:** 2019-12-02

**Authors:** L Knight, M Ranganathan, T Abramsky, T Polzer-Ngwato, L Muvhango, M Molebatsi, H Stöckl, S Lees, C Watts

**Affiliations:** 1 London School of Hygiene and Tropical Medicine, 15–17 Tavistock Place, London, WC1H 9SH UK; 2 Social Surveys Africa, Johannesburg, South Africa; 3 Intervention with Microfinance for AIDS and Gender Equity (IMAGE), Johannesburg, South Africa

**Keywords:** Scaled-up intervention, Intimate partner violence prevention, Microfinance plus, Loan groups, Gender training, South Africa

## Abstract

The Intervention with Microfinance for AIDS and Gender Equity (IMAGE) programme has been scaled up to three provinces in South Africa. This paper explores associations between women’s engagement in the intervention, intimate partner violence (IPV) and factors associated with IPV and partner abuse. We enrolled women receiving group-based microfinance loans plus gender training into the scaled-up IMAGE cohort study (*n* = 860). We present cross-sectional analysis on participants’ characteristics and intervention engagement and use multivariate logistic regression to explore associations. 17% of women reported lifetime (95% CI 15 to 20%) and 7% past year (95% CI 5 to 9%) IPV, 9% past-year economic (95% CI 7 to 11%) and 11% past-year emotional (95% CI 9 to 14%) abuse. Women under 35 years had higher levels of IPV and emotional abuse. 53% of women attended all the trainings, 83% continuously borrowed and 98% agreed the training had a major impact on their life. Attendance was associated with improved partner relationships (*χ*^2^*p* < 0.001), but not lower IPV risk. Odds of past-year IPV decreased the more types of support (e.g. advice) women received from group members (aOR 0.27, *p* < 0.001 among those reporting *all support* versus *none or some*). A similar pattern was seen for economic, but not emotional, abuse. The scaled-up IMAGE intervention is widely acceptable and may support improvements in partner relationships, but younger women need to be targeted. Group support appears to be a potentially important component of the intervention.

**Electronic supplementary material:**

The online version of this article (10.1007/s11121-019-01070-w) contains supplementary material, which is available to authorized users.

## Introduction

Intimate partner violence (IPV) and human immunodeficiency virus (HIV) are major public health challenges in South Africa; however, evidence on large-scale interventions that have successfully addressed both is sparse (Wagman et al. 2015). According to the 2016 South Africa DHS Survey, 21% of ever-partnered women aged 18 and older reported lifetime physical IPV, whilst 8% reported past-year physical IPV; in addition, women reported 6% and 2% lifetime and past-year sexual IPV, respectively. HIV prevalence among South African women aged 15–49 years is estimated at approximately 24% (UNAIDS 2016). In high HIV prevalence settings such as South Africa, HIV infection is highly associated with physical and emotional violence and male controlling behaviour (Durevall and Lindskog 2015).

Poverty, unemployment, lack of economic opportunity and gender inequalities are structural factors that influence both IPV and HIV risk (Gibbs et al. 2017). Microfinance is an approach that increases people’s ability to generate income and secure livelihoods (Kennedy et al. 2014). In addition to the economic benefits of microfinance, there is some mixed evidence to suggest that it may be effective as a means for economically empowering women (Gibbs et al. 2012; Vyas and Watts 2009; de Mel et al. 2009; Miled and Rejeb 2015; Niner 2019.). The idea is acquiring new business skills may enhance their self-esteem, conflict resolution ability and household decision-making power and expand their social networks (Kim et al. 2009).

The Intervention with Microfinance for AIDS and Gender Equity (IMAGE) programme consists of a group lending microfinance initiative with gender and HIV training (Pronyk et al. 2005a, b). In 2006, a cluster randomised trial conducted in Limpopo region of South Africa showed that the IMAGE intervention was associated with a 55% reduction in past-year physical and/or sexual IPV (Pronyk et al. 2006), decreased HIV-related risk behaviours in young women (Pronyk et al. 2008) and improved empowerment indicators (Kim et al. 2009). IMAGE is now operational as a violence prevention programme and has been scaled up in three provinces in South Africa with over 25,000 participating households. This transition to an operational programme offered an opportunity to explore whether the IMAGE programme positively influenced women’s lives, almost a decade on from when it was first designed in the context of a randomised control trial. In this paper, we aim to describe cohort characteristics and women’s engagement with the intervention and explore individual and intervention engagement factors associated with IPV and abuse among rural South African women.

## Methods

### Intervention

IMAGE combines a poverty-focused microfinance initiative implemented by the Small Enterprise Foundation (SEF), with a ten-session participatory curriculum of gender and HIV training known as Sisters for Life (SfL). Loans are administered by SEF for women to develop their own small business. Women attend loan centre meetings and groups of five women guarantee each other’s loans. The gender and HIV training is a compulsory component of the routine loan centre meeting and is run by the SfL team generally over a period of 6 months. The SfL intervention is generally delivered over two phases: phase 1 is *SfL training* that consists of ten 1-h training sessions and covers topics including gender roles, cultural beliefs, relationships, communication, intimate partner violence and HIV, and aims to strengthen communication skills, critical thinking, solidarity and leadership; and phase 2 is *community mobilisation* that encourages collective action to engage both young people and men in the intervention communities. In this setting, phase 2 differs from the original trial design described in detail elsewhere (Hargreaves et al. 2010) by decentralising the community engagement responsibility from a selection of ‘natural leaders’ to all women. It consists of training on community engagement and leadership in four sessions over 5 months following phase 1. This paper describes results from one region, the Mahikeng IMAGE programme, where phase 1 was completed at the time of this survey.

### IMAGE Cohort Study Round-One Survey

The IMAGE cohort study examines changes in loan group women’s vulnerability to IPV over two time points, along with other indicators of economic and social empowerment. We calculated our sample size using McNemar’s test of paired changes in proportions. This was in order to give us an 80% power to detect change (at the 5% significance level) in past-year physical and/or sexual IPV of the same magnitude as seen in the original 2006 IMAGE trial (https://www.statstodo.com/SSizMcNemar_Pgm.php). The required sample size was 852 women (further details available on request). We conducted the round-one survey directly after the loan group women had received the phase 1 SfL training and before the completion of phase 2 community engagement. The follow-up survey was conducted a year later. This analysis reports on the round-one cross-sectional survey data only.

### Study Setting

The study took place in rural Mahikeng region in South Africa’s North West province. We selected this site as SfL were delivering training in this area in 2016. There are 77 loan centres in the Mahikeng area, comprising a total of 460 loan groups (4–8 loan groups per centre) and a total of 2399 loan recipients (approximately 5 women per group). Due to operational reasons, we included 88% (68 of 77) of the SEF centres in the Mahikeng region; no crucial differences in population or intervention delivered were anticipated between centres included and those not included. An average of 15 women were survey interviewed per centre (full range 4 to 27, IQR 11–19).

### Survey Procedures

A South African data collection agency (Social Surveys South Africa (SSA)) co-ordinated and led the fieldwork with London School of Hygiene and Tropical Medicine (LSHTM) researchers providing technical oversight and support. Fifteen female interviewers attended 3 weeks of training on quantitative interviewing techniques and the questionnaire, personal reflections on violence and self-care. All interviewers were trained on violence research ethics, managing disclosures and referral protocols. Interviewer teams were organised into groups of five, including one supervisor. We conducted interviewer-administered survey questionnaires with data captured on to tablet devices. Women were recruited to the study and completed the first-round survey between November and December 2016. All women were offered details of local social and counselling support services at the end of the survey interview.

### Participant Recruitment

We used the following criteria for selecting participants: women 18 years or older, enrolled for a year or more in the Mahikeng branch of SEF loan centres where SfL training sessions were recently completed. We recruited participants from loan meetings, after introducing the study. The research supervisors selected 10 to 20 women who were attending the meeting on that day by pulling names from an opaque bag and invited selected women to participate in the study. The number of women selected depended on the centre size; 20 in large centres, 15 in small centres, if fewer women were present we invited all to consent. If a woman was unable or unwilling to stay, or refused to consent, the reason was documented. All women were provided with mobile phone airtime worth R50 (4 USD) immediately after the survey interview.

### Data Management

All identifying information was recorded and stored separately from questionnaire responses. Questionnaires were assigned a unique ID. Tablet devices were programmed with logical checks and skips, and data were uploaded directly to a secure server using KoBo Toolkit (www.kobotoolbox.org). Data were managed in excel and Stata14 (StataCorp 2015), and missing or inconsistent data queried with participants, as per the data quality protocol. Data were missing in less than 1% of cases for the majority of variables used in analysis. Records with missing data were not included in summary statistics or relevant regression models.

### Study Measures

#### Violence and Abuse Outcome Measures

Physical, sexual and emotional IPV questions were adapted from the WHO Multi-country Study on Women’s Health and Domestic Violence Against Women survey questions and translated to the local language, Tsetswana. Women who had a partner in the last year were asked about acts of sexual and physical violence and economic abuse experienced in the last 12 months. The definition of a partner included currently married or currently living with a man as if married, partner but not living together, casual boyfriends and any type of partner in the last 12 months (among women not in relationship). Emotional abuse and controlling behaviour questions were adapted from the What Works violence prevention programme (http://www.whatworks.co.za/about/about-what-works) in South Africa. Binary violence outcome variables were constructed with positive responses to one or more violent acts coded as 1 and all others coded as 0. A full list of violence outcome questions is shown in Table 1.

**Table 1:**
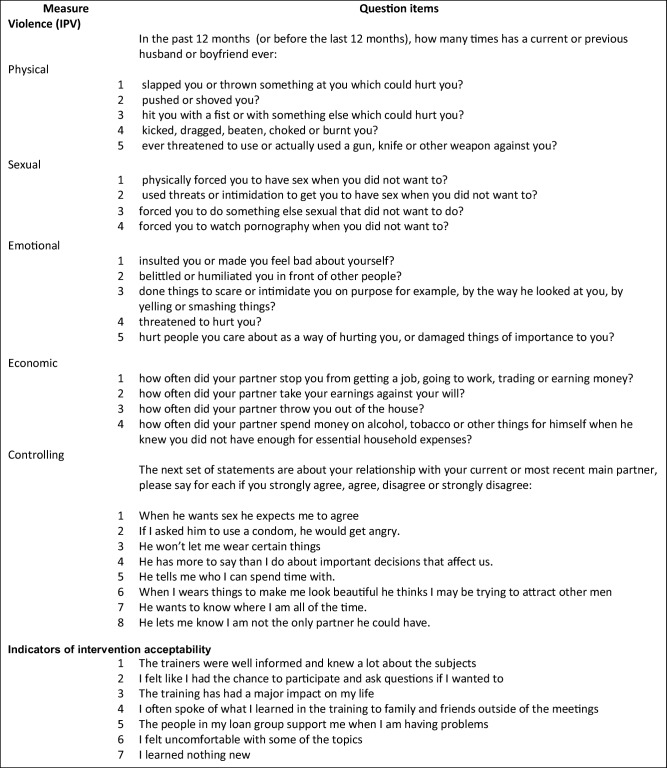
Violence questions and indicators of intervention acceptability

#### IMAGE Intervention Engagement Measures

Intervention uptake measures include “Loan borrowing” categorised as continuously borrowed, interrupted or new loan in the last year; “SfL training attendance” categorised as all, half or more and less than half of the training attended. The intervention experience measures include the following: acceptability questions listed in Table. 1 (Hargreaves et al. 2010), with summary measures “Positive about the training and group” and “support provided by the group” shown in Annex 1 in the ESM; and “Microfinance loan influence on relationship” where women were asked how being a member of a microfinance group had influenced their relationship with their partner over the last 12 months, with the following response options: did not affect the relationship, made your relationship more difficult, improved your relationship, not in a relationship. A full description of intervention engagement measures is provided in Annex 1 in the ESM.

#### Other Constructed Measures

We constructed socio-economic status using variables that capture living standards, such as household ownership of durable assets (e.g. TV, fridge) and infrastructure and housing characteristics (e.g. source of water, sanitation facility). We used principle component analysis on asset data to derive a socio-economic status index and then grouped households into categories reflecting different socio-economic status levels (Vyas and Kumaranayake 2006; Ranganathan et al. 2019)

### Analysis

All analysis presented is exploratory. Sociodemographic characteristics and prevalence of IPV and abuse are described by number and percentage and compared across age groups with associated *χ*^2^*p* values (Table 2). Cross-tabulations between women’s characteristics and intervention factors are presented along with *χ*^2^*p* values (Table 3). To explore associations with lifetime and past-year IPV and abuse, we fitted a series of logistic regression models. To differentiate risk factors for past-year IPV from those associated with IPV experienced before the last year, we removed those who had experienced IPV prior to the past year only from the denominator (Abramsky et al. 2011; Durevall and Lindskog 2015).

**Table 2:** Sociodemographic characteristics, sexual behaviour and partner violence and abuse, across age groups

	Total sample	Age group			
		21–34	35–54	55–82	
	*n* (%)	*n* (%)	*n* (%)	*n* (%)	*p* value
Sociodemographic characteristics					
Total cohort	860	145 (16.9)	409 (47.6)	305 (35.5)	
Marital status					
Currently married/living as married	415 (48.2)	61 (42.1)	223 (54.8)	127 (42.3)	< 0.001
Divorced/relationship ended	113 (13.2)	7 (4.8)	59 (14.4)	47 (15.4)	
Widowed	156 (18.2)	0	51 (12.5)	102 (34.4)	
Never married	176 (20.5)	77 (53.1)	75 (18.3)	24 (7.9)	
Level of education ^a^					
Primary or lower	334 (39.7)	21 (14.6)	138 (34.8)	175 (58.3)	< 0.001
Secondary (any grade)	400 (47.6)	89 (61.8)	203 (51.1)	108 (36.0)	
Passed matric	87 (10.4)	28 (19.4)	48 (12.1)	11 (3.7)	
Further education	20 (2.4)	6 (4.2)	8 (2.0)	6 (2.0)	
Number of children < 18 living at home					
0 children	217 (25.3)	19 (13.1)	69 (16.9)	129 (42.3)	< 0.001
1–2 children	363 (42.21)	51 (35.2)	194 (47.4)	118 (38.7)	
3+ children	280 (32.6)	75 (51.7)	146 (35.7)	58 (19.0)	
Female-headed household	456 (53.02)	67 (46.2)	205 (50.1)	184 (60.3)	0.005
Household assets					
Low	172 (20.0)	38 (26.2)	89 (21.8)	45 (14.8)	0.031
Medium	512 (59.9)	83 (57.6)	247 (60.5)	182 (60.1)	
High	172 (20.0)	24 (16.6)	75 (18.3)	73 (23.9)	
Personal earning during the past 12 months	587 (68.3)	90 (62.1)	291 (71.2)	206 (67.5)	0.121
Monthly household income ^a^					
R2000 or less ($142 USD or less)	207 (24.3)	33 (22.9)	98 (24.2)	76 (25.1)	0.052
R2001 to R3500	231 (27.00)	37 (25.69)	100 (24.69)	93 (30.69)	
R3501 to R5000	180 (21.13)	27 (18.25)	83 (20.49)	70 (23.10)	
R5001 to R8000	136 (15.96)	23 (15.97)	69 (17.04)	44 (14.52)	
Over R8000 (over $571 USD)	99 (11.6)	24 (16.7)	55 (13.6)	20 (6.6)	
Sexual behaviour					
Sexual debut ^a^					
18 years or older	608 (75.0)	100 (69.0)	298 (72.9)	254 (83.3)	0.019
15 to 17 years old	166 (20.4)	36 (26.0)	90 (22.7)	40 (14.4)	
Under 15 years old	41 (5.0)	9 (6.4)	21 (5.3)	11 (4.0)	
Condom use at last sex with main partner ^b^	294 (35.6)	69 (47.6)	164 (41.1)	61 (21.6)	< 0.001
Starting or staying in a relationship in the last 12 months to receive monetary benefits	55 (6.4)	19 (13.1)	25 (6.1)	11 (3.6)	0.001
Two or more sex partners in last 12 months	78 (9.1)	25 (17.2)	44 (10.8)	9 (3.0)	< 0.001
Partner violence and abuse, lifetime					
Physical violence	128 (14.9)	38 (26.2)	57 (13.9)	33 (10.8)	< 0.001
Sexual violence	65 (7.6)	9 (6.2)	38 (9.3)	18 (5.9)	0.189
Physical and/or sexual violence	146 (17.0)	41 (28.3)	71 (17.4)	34 (11.2)	< 0.001
Economic abuse	123 (14.3)	29 (20.0)	59 (14.4)	35 (11.5)	0.054
Partner violence and abuse, last 12 months					
Physical violence	46 (5.4)	25 (17.2)	16 (3.9)	5 (1.6)	< 0.001
Sexual violence	25 (2.9)	7 (4.8)	10 (2.4)	8 (2.6)	0.318
Physical and/or sexual violence	57 (6.6)	27 (18.6)	20 (4.9)	10 (3.3)	< 0.001
Economic abuse	77 (9.0)	21 (14.5)	38 (9.3)	18 (5.9)	0.011
Emotional abuse	96 (11.2)	33 (22.8)	45 (11.0)	18 (5.9)	< 0.001
Partner controlling behaviour, in current or most recent relationship	448 (52.1)	98 (67.6)	234 (57.2)	115 (37.7)	< 0.001
Partner violence, abuse or control, life time	500 (58.2)	110 (75.9)	257 (62.8)	133 (43.6)	< 0.001

Missing data: ^a^1 age, 5 (1%) household SES, 8 (1%) household income, 9 (1%) number in household, 19 (2%) education, 44 (5%) sexual debut; ^b^*n* = 826 as 18 refused to answer and 15 said they had not had sex since the first time

**Table 3:** Associations between women’s characteristics, intervention uptake and microfinance group membership influence on relationship

	*N*	Microfinance loan borrowing			*p* value	SfL training attendance ^a^				*N*	Microfinance group influence on relationship^b^			
		Continuously	New loan	Interrupted		All	Half or more	Less than half	*p* value		More difficult	Not affected	Improved	*p* value
		%	%	%		%	%	%			%	%	%	
Total, number (%)	860	712 (82.8)	112 (13.0)	35 (4.1)		455 (53.0)	281 (32.7)	117 (13.6)		581	15 (2.6)	304 (52.3)	262 (45.1)	
Age														
< 35	144	74%	21%	4%	< 0.01	41%	37%	21%	0.001	125	3%	60%	37%	0.13
35–55	409	83%	12%	5%		51%	36%	13%		309	3%	52%	45%	
> 55	305	87%	10%	3%		61%	27%	12%		146	1%	47%	52%	
Education														
Primary or lower	334	85%	13%	3%	0.38	54%	32%	14%	0.57	198	3%	54%	44%	0.71
Secondary	400	81%	14%	5%		53%	31%	15%		299	2%	52%	46%	
Higher	107	86%	19%	4%		46%	39%	15%		73	4%	55%	41%	
Household assets														
Lowest	172	77%	19%	3%	0.09	48%	33%	20%	0.22	108	2%	62%	36%	0.17
Medium	512	84%	11%	4%		54%	34%	13%		345	2%	51%	46%	
Highest	172	84%	13%	3%		56%	31%	13%		127	4%	47%	49%	
Number children at home														
None	217	83%	14%	4%	0.99	61%	26%	13%	0.05	124	2%	52%	45%	0.45
1 or 2	362	83%	13%	4%		52%	34%	14%		256	3%	48%	48%	
3 or more	280	83%	13%	4%		48%	37%	15%		201	2%	57%	41%	
Marriage status														
Never	172	79%	15%	6%	0.39	39%	43%	18%	< 0.01	117	3%	68%	29%	< 0.001
Divorced	113	85%	13%	2%		56%	28%	16%		27	0%	74%	26%	
Widowed	156	88%	9%	3%		57%	29%	14%		30	1%	70%	27%	
Currently married	415	82%	14%	4%		57%	31%	12%		407	2%	45%	52%	
SfL training attended														
All	455	88%	9%	3%	< 0.001	-	-	-		295	2%	43%	56%	< 0.001
Half or more	282	85%	9%	6%		-	-	-		197	4%	56%	40%	
Less than half	122	60%	36%	4%		-	-	-		89	3%	75%	21%	
Loan borrowing														
Continuously	-	-	-	-		-	-	-		470	2%	51%	50%	0.18
New loan	-	-	-	-		-	-	-		82	4%	56%	40%	
Interrupted	-	-	-	-		-	-	-		28	7%	61%	32%	

*p* value: *χ*^2^*p* value unless 1 or more cells with frequency of 5 or less, in which case Fisher exact *p* value presented

^a^Six women (0.7%) said they had attended none of the training, these women have been grouped with less than half

^b^In past 12 months, how has being a member of a microfinance group influenced your relationship with your partner? 129 said they were not in a relationship

Sociodemographic and sexual behaviour factors shown to be important predictors of IPV and abuse in other settings were selected for bivariate analysis (Abramsky et al. 2011). Multivariate logistic regression models were fitted using factors crudely associated with each outcome at a *p* value of < 0.05, as well as age and household assets, which were identified a priori (Table 6). Finally, we present associations between intervention engagement and IPV and abuse experienced in the last 12 months. Crude and adjusted odds ratios are shown, with final model adjusted by all intervention engagement factors, age group and household assets selected a priori (Table 7). In the multivariate regression exploring intervention engagement factors associated with past-year IPV and abuse, we performed sensitivity analyses to explore marriage status, education level and number of children living at home as potential confounding factors.

## Results

### Participants

A total of 937 eligible women attending loan groups were selected and invited to consent to the study, of whom 860 (92%) consented and completed a cross-sectional survey. Reasons for not consenting included the following: health reasons, going to post office to make loan payment, going to work, school or funeral, and no time.

### Participant Characteristics and Comparisons Across Age Groups

The median age of the women was 48 years, with over a third aged over 55 years. Almost half were currently married or living as if married, 40% did not reach secondary school education and a quarter reported a monthly household income of less than $142 USD. Younger women, defined as under 35 years old throughout this manuscript, lived in households with fewer assets and had more children living at home. HIV risk behaviours varied across age groups; older women reported less condom use, whilst younger women reported earlier sexual debut, more partners in the last 12 months and a greater likelihood of having started or stayed in a relationship in the last year to receive monetary benefits.

### Prevalence of Partner Violence and Abuse

Lifetime and past-year prevalence of partner physical and/or sexual violence for the entire cohort was 17% (95% CI 15 to 20%) and 7% (95% CI 5 to 9%), respectively (Table 2). The past-year prevalence of economic and emotional partner abuse was 9% (95% CI 7 to 11%) and 11% (95% CI 9 to 14%), respectively. Controlling behaviour in current/most recent relationship was reported by 53% (95% CI 49–56%) of women.

### Intervention Uptake and Influence on Relationship

Table 3 presents cross-tabulations of women’s sociodemographic characteristics and self-reports of loan borrowing, SfL training attendance and influence of the microfinance loans on relationships. Most women borrowed continuously over the last year; however, 13% started a new loan, and 4% had interrupted borrowing. The most common reason given for interrupted borrowing was the inability to personally pay back loans or due to group payment issues; other reasons were personal issues such as sickness or travel and business issues such as “not selling”. Over half attended all of the training sessions, a third attended more than half but not all and the remaining 14% attended less than half, including six women who attended none. Of the women who said they were in a current relationship, 3% said that being a member of a microfinance group made their relationship more difficult, 52% said it had not affected their relationship and 45% said it had improved their relationship.

Younger women, those who attended less SfL training and those in the lowest socio-economic status households, were more likely to report having started a new loan in the past year. Younger women, those currently unmarried nor living as married and those who had children living with them, reported attending less SfL training. Greater training attendance was associated with women who were more likely to report improved relationships with their partner, with women attending all the sessions being the most likely to report an improvement and the least likely to report that relationship had become more difficult. Younger women, those with fewer household assets and those who interrupted loan borrowing, were less likely to report an improvement in their relationship; however, these associations did not reach statistical significance.

### Intervention Acceptability and Group Support

Table 4 shows that the majority of women were positive about the intervention. Most women reported that members of their group provided the various types of support pertaining to financial, business, personal and emotional concerns; however, material support such as clothes and food was reported to a much lesser extent.

**Table 4:** Indicators of intervention acceptability

Indicators of acceptability	I strongly agree	I agree	I disagree	I strongly disagree
	*n* (%)	*n* (%)	*n* (%)	*n* (%)
Positive about training and group:				
1. The trainers were well informed and knew a lot about the subjects	731 (85.6)	114 (13.4)	5 (0.6)	4 (0.5)
2. I felt like I had the chance to participate and ask questions if I wanted to	583 (68.3)	236 (27.6)	23 (2.7)	12 (1.4)
3. The training has had a major impact on my life	641 (75.1)	202 (23.7)	5 (0.6)	6 (0.7)
4. I often spoke of what I learned in the training to family and friends outside of the meetings	637 (74.6)	180 (21.1)	21 (2.5)	16 (1.9)
5. The people in my loan group support me when I am having problems	606 (71.0)	203 (23.8)	33 (3.9)	12 (1.4)
Views on the training:				
6. I felt uncomfortable with some of the topics	204 (23.9)	115 (13.5)	221 (25.9)	314 (36.8)
7. I learned nothing new	192 (22.8)	27 (3.1)	130 (15.2)	502 (58.8)

Table 5 shows intervention summary measures. Just under half the women were highly positive about all aspects of the training and over half of the women reported that all types of support were provided by the group members. Notably, there was no difference between age groups with regard to a positive attitude about the training, engagement with the topics or support provided from the group (Annex 1 in the ESM).

**Table 5:** Types of support provided by group members

Types of support provided by group members	Yes, *n* (%), *n* = 860
Help with financial issues	774 (90.0)
Advice with business issues	781 (90.8)
Advice with personal issues	744 (86.5)
Other material support (such as food or clothing)	471 (54.8)
Emotional support (love, caring, friendship)	763 (88.7)

### Individual Factors Associated with Intimate Partner Violence and Abuse

Table 6 presents associations between women’s sociodemographic factors and both lifetime and past-year experiences of IPV and abuse. Women aged over 55 had lower odds, and women who had earned money in the last year had higher odds, of lifetime sexual and/or physical IPV. Women reporting earlier sexual debut had higher odds of lifetime and double the odds of past-year sexual and/or physical IPV, compared with women whose sexual debut occurred at 18+ years of age. Younger women had a much greater risk of recent IPV and recent emotional abuse with older age groups having approximately a third the odds of sexual and/or physical IPV and emotional abuse in the last year. Women who reported two or more sexual partners in the past year had almost three times the odds of recent emotional abuse compared to those with fewer partners.

**Table 6:** Factors associated with lifetime and recent partner violence and abuse

	Partner physical and/or sexual violence		Partner physical and/or sexual violence		Partner emotional abuse		Partner economic abuse		Partner economic abuse	
	Lifetime		Last 12 months^a^		Last 12 months		Lifetime^b^		Last 12 months^a,b^	
	OR 95% CI *p* value	aOR 95% CI *p* value	OR 95% CI *p* value	aOR 95% CI *p* value	OR 95% CI *p* value	aOR 95% CI *p* value	OR 95% CI *p* value	aOR 95% CI *p* value	OR 95% CI *p* value	aOR 95% CI *p* value
Total number in model	859	848	771	749	860	837	859	848	814	791
Age group										
< 35 years	ref	ref	ref	ref	ref	ref	ref	ref	ref	ref
35–55 year	0.53 0.34–0.83 0.01	0.53 0.36–0.96 0.03	0.23 0.12–0.42 < 0.001	0.35 0.18–0.68 0.002	0.42 0.26–0.69 0.001	0.52 0.30–0.90 0.02	0.67 0.41–1.10 0.12	0.79 0.46–1.36 0.40	0.60 0.34–1.06 0.08	0.87 0.46–1.65 0.67
55+ years	0.32 0.19–0.53 < 0.001	0.42 0.23–0.77 0.01	0.14 0.07–0.30 < 0.001	0.34 0.14–0.88 0.03	0.21 0.12–0.39 < 0.001	0.38 0.97–2.87 0.06	0.52 0.30–0.89 0.02	0.82 0.43–1.55 0.54	0.37 0.19–0.72 0.01	1.00 0.43–2.30 0.57
Household assets										
Low	ref	ref	ref	ref	ref	ref	ref	ref	ref	ref
Medium	0.84 0.57–1.27 0.42	0.80 0.50–1.29 0.37	0.84 0.44–1.59 0.59	0.75 0.37–1.51 0.42	1.10 0.63–1.88 0.77	0.96 0.53–1.73 0.89	0.82 0.51–1.31 0.40	0.71 0.43–1.16 0.17	0.65 0.37–1.13 0.13	0.52 0.28–0.97 0.04
High	1.00 0.59–1.73 0.80	1.12 0.62–1.99 0.71	0.50 0.20–1.29 0.15	0.47 0.17–1.31 0.15	0.83 0.41–1.68 0.61	0.73 0.34–1.58 0.43	0.71 0.38–1.27 0.23	0.56 0.29–1.06 0.08	0.64 0.31–1.30 0.21	0.51 0.23–1.13 0.10
Marriage status										
Never married	ref	ref	ref	ref	ref	ref	ref	ref	ref	ref
Divorced/separated	0.60 0.33–1.11 0.11	0.75 0.38–1.45 0.38	0.13 0.03–0.59 0.01	0.24 0.05–1.12 0.07	0.58 0.26–1.29 0.18	1.06 0.44–2.57 0.89	0.52 0.30–0.92 0.02	0.67 0.36–1.28 0.23	0.09 0.03–0.33 < 0.001	0.16 0.04–0.60 0.01
Widowed	0.31 0.16–0.60 < 0.001	0.47 0.23–0.98 0.04	0.04 0.01–0.34 < 0.001	0.13 0.02–1.04 0.05	0.09 0.02–0.37 < 0.001	0.23 0.05–1.08 0.06	-	-	-	-
Married/living as if married	0.67 0.44–1.03 0.07	0.76 0.47–1.22 0.26	0.61 0.34–1.10 0.10	0.72 0.32–1.60 0.18	1.17 0.70–1.95 0.55	1.21 0.61–2.42 0.59	0.94 0.58–1.50 0.79	0.83 0.44–1.58 0.58	1.21 0.70–2.12 0.50	1.35 0.64–2.86 0.43
Level of education										
Primary or lower	ref	ref	ref	ref	ref	ref	ref	-	ref	ref
Secondary (any grade)	1.51 1.02–2.24 0.04	1.17 0.76–1.79 0.49	2.21 1.14–4.29 0.02	1.60 0.77–3.33 0.21	2.07 1.25–3.42 0.01	1.66 0.97–2.86 0.07	1.48 0.98–2.14 0.07	-	2.09 1.21–3.60 0.01	2.0 1.11–3.70 0.02
Pass metric	1.38 0.74–2.58 0.31	0.93 0.76–1.79 0.82	3.12 1.31–7.41 0.01	1.93 0.74–5.04 0.18	2.07 0.99–4.33 0.05	1.58 0.71–3.51 0.92	0.80 0.38–1.72 0.58	-	1.50 0.64–3.54 0.35	1.33 0.52–3.40 0.55
Further education	0.68 0.15–3.03 0.61	0.40 0.08–1.91 0.25	1.23 0.15–9.94 0.85	0.92 0.09–9.21 0.94	1.44 0.32–6.57 0.64	0.92 0.18–4.77 0.92	0.37 0.05–2.81 0.34	-	0.77 0.10–6.06 0.81	1.08 0.12–9.88 0.95
Two or more sexual partners in 12 months	1.41 0.80–2.50 0.24	-	2.28 1.10–4.73 0.03	1.48 0.67–3.31 0.12	3.47 2.00–6.04 < 0.001	2.74 1.52–4.95 < 0.001	1.35 0.73–2.49 0.34	-	2.14 1.12–4.09 0.02	1.69 0.83–3.43 0.15
Sexual debut										
18 years and over	ref	ref	ref	ref	ref	-	ref	ref	ref	ref
15 to 17 years old	1.82 1.20–2.76 0.01	1.77 1.14–2.73 0.01	2.14 1.16–3.95 0.01	2.10 1.10–4.10 0.03	1.43 0.86–2.38 0.17	-	1.91 1.23–3.00 < 0.001	1.80 1.17–2.94 0.01	1.84 1.14–2.83 0.01	1.95 1.09–3.49 0.03
Under 15 years old	1.61 0.75–3.50 0.22	1.69 1.14–2.73 0.20	2.48 0.91–6.76 0.08	2.42 0.80–7.30 0.12	1.83 0.78–4.29 0.16	-	1.74 0.77–3.90 0.18	1.80 0.78–4.11 0.17	1.79 0.83–5.21 0.12	2.74 0.98–7.68 0.06
Female household head	0.71 0.50–1.01 0.06	-	0.45 0.26–0.80 0.01	0.59 0.27–1.27 0.18	0.38 0.24–0.60 < 0.001	0.55 0.29–1.05 0.07	0.65 0.44–0.96 0.03	0.73 0.41–1.31 0.29	0.40 0.24–0.66 < 0.001	0.73 0.37–1.47 0.38
Number of children living with you										
None	ref	-	ref	-	ref	-	ref	ref	ref	ref
1 or 2	1.09 0.69–1.72 0.72	-	1.51 0.71–3.22 0.28	-	1.16 0.66–2.02 0.61	-	1.69 1.00–2.84 0.05	1.55 0.90–2.68 0.11	2.77 1.31–5.85 0.01	2.25 1.01–5.00 0.05
3 or more	1.20 0.75–1.93 0.46	-	1.76 0.81–3.81 0.15	-	1.33 0.75–2.36 0.33	-	1.61 0.93–2.98 0.09	1.36 0.75–2.44 0.31	2.65 1.23–5.73 0.01	2.36 1.02–5.52 0.05
Personally earned in last 12 months	1.66 1.10–2.51 0.02	1.77 1.14–2.73 0.01	1.29 0.71–2.35 0.41	-	1.08 0.68–1.72 0.73	-	1.00 0.66–1.51 0.99	-	0.59 0.37–0.95 0.03	0.65 0.39–1.08 0.09
Monthly household earning										
$142 or less	ref	-	ref	-	ref	-	ref	ref	ref	-
$142–$357	1.08 0.69–1.71 0.73	-	1.18 0.60–2.31 0.64	-	1.23 0.72–2.10 0.46	-	1.42 0.85–2.37 0.17	1.32 0.78–2.24 0.31	1.15 0.62–2.14 0.65	-
$357–$571	1.23 0.69–2.19 0.47	-	0.85 0.33–2.19 0.74	-	1.02 0.50–2.08 0.96	-	1.14 0.59–2.23 0.70	1.01 0.50–2.04 0.33	1.16 0.53–2.54 0.71	-
Over $571	1.38 0.69–2.43 0.41	-	1.18 0.45–3.1 0.74	-	1.00 0.45–2.20 0.99	-	2.15 1.13–4.12 0.02	1.88 0.93–3.81 0.08	2.1 0.96–4.43 0.06	-

^a^Women experiencing violence before the last 12 months only have been removed from the denominators for physical and/or sexual violence (*n* = 89) and economic abuse (*n* = 46), past violence was not captured for emotional abuse

^b^Widowed, divorced or separated grouped together in the economic abuse models

^c^Logical rule (Gelman and Hill 2007) applied for sexual debut missing values—this pertained mostly to older women who “don’t remember” and we thus included them in the reference group in regression models. Notation: *aOR* adjusted odds ratio (adjusted for all other covariates in the model), *95% CI* confidence interval

Earlier sexual debut was consistently associated with partner economic abuse, whilst, women’s current age was not associated with economic abuse, in the adjusted models. Living in a house with more assets seems to be protective, with women in households with medium or high number of assets having half the odds of abuse compared with those with the least number of assets. Women who attended secondary school had double the odds of economic abuse compared with those with less education attainment and having children living at home is associated with more than double the odds of experiencing past-year economic abuse.

### Intervention Engagement Association with Intimate Partner Violence and Abuse

Table 7 presents logistic regression crude and adjusted odds ratios showing associations between intervention factors and past-year IPV and abuse. Compared with continuous borrowing, interrupted loan borrowing over the last year was associated with three times higher odds of past-year emotional abuse (aOR 3.02, *p* = 0.01), whereas starting a new loan in the last year was associated with higher odds of past-year economic abuse (aOR 2.62, *p* ≤ 0.001). Attending half or more, but not all, of the training sessions was associated with reporting less emotional abuse in the last year compared with those women attending all the sessions (aOR 0.52, *p* = 0.02). Women reporting all types of support provided from the group, compared with none or some support, had almost a quarter the odds of past-year physical and/or sexual violence (aOR 0.27, *p* ≤ 0.001). Similarly, most types of support provided (aOR 0.38, *p* = 0.01) and all types of support provided (aOR 0.41, *p* = 0.01) from the group members was associated with reduced odds of women reporting past-year economic abuse. The sensitivity analysis, which included additionally adjusting for marriage status, number of children living at home and education attainment, produced similar results.

**Table 7:** Associations between IMAGE programme engagement and past-year partner violence and abuse

		Physical or sexual violence		Emotional abuse		Economic abuse	
		Last 12 months		Last 12 months		Last 12 months	
	n (%)	OR 95% CI *p* value	aOR 95% CI *p* value	OR 95% CI *p* value	aOR 95% CI *p* value	OR 95% CI *p* value	aOR 95% CI *p* value
Women’s engagement with the IMAGE programme							
Number in model	860	771	765	860	854	814	808
Loan borrowing, in last 12 months							
Continuously	712 (82.9)	ref	ref	ref	ref	ref	ref
New loan	112 (13.0)	1.29 0.61–2.72 0.51	0.88 0.38–2.04 0.78	1.90 1.10–3.31 0.02	1.78 0.97–3.26 0.06	2.91 1.68–5.01 < 0.001	**2.62** **1.44–4.77** **< 0.001**
Interrupted	35 (4.1)	2.54 0.93–6.93 0.07	2.13 0.71–6.38 0.18	2.76 1.21–6.31 0.02	*3.05* *1.26–7.38* *0.01*	0.73 0.17–3.11 0.67	0.60 0.14–2.63 0.50
Training attendance							
All	455 (52.9)	ref	ref	ref	ref	ref	ref
Half or more	282 (32.8)	0.82 0.43–1.56 0.54	0.53 0.27–1.15 0.07	0.68 0.41–1.11 0.13	*0.52* *0.31–0.89* *0.02*	1.12 0.66–1.89 0.68	0.97 0.56–1.69 0.92
Less than half	123 (14.3)	1.64 0.81–3.30 0.17	1.07 0.47–2.44 0.87	0.90 0.48–1.67 0.73	0.58 0.29–1.16 0.12	1.32 0.68–2.57 0.41	0.76 0.36–1.59 0.46
Positive about the training and group							
High (count:20)	372 (43.3)	ref	ref	ref	ref	ref	ref
Medium (count: 17–19)	244 (40.0)	0.77 0.41–1.43 0.40	0.70 0.35–1.37 0.30	0.94 0.59–1.52 0.81	0.99 0.60–1.64 0.97	1.30 0.77–2.17 0.34	1.09 0.63–1.89 0.77
Low (count: 5–16)	144 (16.7)	1.25 0.62–2.51 0.53	0.93 0.42–2.10 0.85	1.23 0.69–2.20 0.49	1.25 0.66–2.35 0.97	1.25 0.64–2.44 0.52	0.93 0.44–1.93 0.84
Types of support provided by group members							
None or some types (0–3 types)	141 (16.4)	ref	ref	ref	ref	ref	ref
Most types (4 types)	271 (31.5)	0.56 0.28–1.11 0.10	0.50 0.24–1.10 0.07	0.80 0.43–1.48 0.48	0.90 0.46–1.73 0.75	0.38 0.20–0.71 < 0.001	*0.38* *0.19–0.74* *0.01*
All types (5 types)	448 (52.1)	0.32 0.16–0.63 0.001	*0.27* *0.12–0.59* *< 0.001*	0.75 0.43–1.33 0.33	0.83 0.44–1.56 0.57	0.39 0.22–069 < 0.001	*0.41* *0.23–0.76* *0.01*

*Adjusted odds ratio* adjusted by women’s age, household asset quintile and all other intervention factors in multivariable model, *95% CI* 95% confidence interval

## Discussion

### Intervention Delivery and Acceptability at Scale

The Mahikeng IMAGE operational programme retained the same original trial intervention components of SEF group loans and phase 1 SfL training topics; however, the phase 2 community mobilisation component had not been delivered at the time of round-one survey. In our cohort, 24% of women reported a monthly household an income of < $142/R2000, and 72% reported less than the North West province average (based on exchanged rates in 2016 and the South African Census 2011). This indicates that, as intended, the operational intervention is reaching the poorest women in the province. Among our sample of women attending loan group meetings, most had continuously borrowed over the last year (83%) and attended half or more of the SfL training (86%). Women’s responses to the operational intervention acceptability questions were similar, or higher, than reported by those women participants in the original trial and immediate scale-up (Hargreaves et al. 2010).

### Younger Women, Partner Violence and Intervention Engagement

Similar to other Sub-saharan African settings, younger age < 35 years old is associated with physical and/or sexual IPV and emotional partner abuse in our cohort (Stöckl et al. 2014; Kapiga et al. 2017). This age group also differed in terms of programme uptake. As observed in the IMAGE trial setting, younger women attended less of the SfL training (Hargreaves et al. 2010). This suggests specific obstacles may affect younger women attendance across settings, for example, pressures looking after young children. The SfL training is a mandatory part of loan group meetings; hence, attendance could be affected by ability to make repayments. Further research to understand challenges that affect younger women’s attendance and efforts to remove barriers are needed.

Intervention acceptability and group support was similar across ages; however, fewer younger women said being part of a microfinance group had a positive effect on their relationship. HIV risk factors, such as more sexual partners, transactional sex and partner controlling behaviours, were reported more by younger women in our cohort, as in national surveys. IMAGE is one of few microfinance plus interventions that has demonstrated reductions in HIV risk behaviours among younger women < 35 (Pronyk et al. 2008; Cui et al. 2013). However, younger women are classed as a credit risk with more out-migration (Kim et al. 2007), as indicated by the higher drop-out rate during the IMAGE trial among this age group (Hargreaves et al. 2010). Therefore, standard microfinance practice remains as targeting women > 35 (Pronyk et al. 2005a, b). Given the potential dual benefit of this intervention to reduce HIV and IPV risk, it might be time to re-evaluate operational risks versus the benefits of targeting women under 35 years in some contexts.

### Intervention Engagement and Partner Violence

Women who had interrupted loan borrowing reported higher odds of IPV and emotional abuse. This could be related to IPV and abuse affecting women’s ability to pay back loans, which has important implications for successful programme delivery, especially as this may indicate a greater issue affecting those who drop out. Further, the associations between starting a new loan and emotional and economic partner abuse may reflect pre-existing partner abuse or conversely indicate increased vulnerability to abuse among those newly joining the programme. This finding suggests a need to equip women from the outset with the skills to deal with potential financial conflict that may arise when first receiving new loans. This supports microfinance plus training interventions for IPV prevention, rather than microfinance alone, whist indicating that specific areas of financial conflict, or conflict arising from the woman’s improved economic position, are still not addressed strongly enough even in such microfinance plus training programmes.

In terms of SfL training, attending all the training sessions, compared with attending half or more, was associated with increased odds of past-year emotional abuse. As seen in other violence prevention programs, it might be that women’s own experience of violence motivates them to engage more with the intervention, potentially masking a positive effect of attending all training on IPV (Abramsky et al. 2014). This could suggest that in later sessions the topic of IPV can be addressed in even more depth, also discussing highly sensitive issues such as sexual IPV that is harder to address (Abramsky et al. 2014). Irregular attenders were likely absent from the loan group during survey sampling and therefore had very low intervention engagement; thus, the association with IPV could not be established. Notably, few women in relationships said that being part of a microfinance loan group made their relationship more difficult and there is an encouraging trend between training attendance and women reporting a positive influence on their relationship. However, it is important to bear in mind that women who perceived the training as having a positive effect on their relationship may be more likely to attend, as opposed to the other way round.

In our study, women who reported more support from group members also reported less past-year IPV and economic abuse. Loan groups with participatory gender training might provide the essential social support that improves self-esteem and emboldens women to challenge existing or new relationship power imbalances and have the confidence to leave abusive relationships (Brody et al. 2017). Hence, improving social capital, in conjunction with economic empowerment, is a potential mechanism that augments reductions in IPV. This finding could point towards the intervention reducing IPV through group support, although the reverse direction of effect is equally as likely. It is possible that women experiencing violence have less confidence to engage with the group and therefore receive lower levels of group support. However, this supports the original IMAGE trial’s qualitative finding that reductions in IPV were brought about in a range of ways, including the provision of material and moral support to those experiencing abuse (Kim et al. 2007). We found that support was similar across household assets levels and age groups; however, women who had never been married reported less group support (Annex 2 in the ESM). Therefore, building group cohesion to support unmarried women is one area of potential intervention development, along with further research on promoting cohesion, such as how groups are formed, the importance of engaging in a group with peers and the frequency of meetings. In addition, further qualitative exploration to unpack the mechanisms by which group support might help protect against IPV in different types of relationships is needed.

### Strengths and Limitations

The study strengths include a high response rate, inclusion of older women and extensive interviewer training emphasising research independence and confidentiality. Further, this is one of few studies of a scaled-up economic empowerment intervention. The study utilised loan group meetings to recruit women and therefore, women with irregular attendance due to factors, such as difficulties making loan repayment or issues at home including IPV, are likely to be under-represented in this sample. Violence in general is underreported, therefore likely underestimated in this study even though we used the WHO current gold standard questions (Abramsky et al. 2011). Further research is required to understand if those experiencing severe IPV have substantial challenges to engage in microfinance and microfinance plus programmes. Encouragingly, the MAISHA trial in Tanzania indicates that women participate in microfinance despite very high levels of violence including severe violence (Kapiga et al. 2017). Another key limitation is the cross-sectional design and therefore, no assertions of causality or direction of effects can be made. Results from the follow-up survey will allow further examination of the temporality of associations, as well as of the persistence of associations over time. There is also a need for additional analysis by severity and frequency of violence and abuse. Sensitivity analysis (results available on request) that modelled intervention engagement association with *any* type and *all* types of past-year violence yielded effects in the same direction as results presented. There is also no control group in this analysis, limiting our capacity to assess intervention impact. However, the 2006 randomised controlled trial has shown IMAGE to be an effective IPV prevention intervention. This study presents associations between IMAGE intervention engagement and IPV after delivery of phase 1, whilst the influence of the phase 2 community mobilisation component will be explored at 1-year follow-up.

### Summary of Findings and Recommendations

Our study found that the scaled-up IMAGE operational programme is widely acceptable among this population of women in rural South Africa. Women who attended more of the training were most likely to report that the intervention had a positive influence on their relationship. However, younger women < 35 years were among the least likely to attend all the >training and report relationship improvements. Younger women are at high risk of IPV and HIV and both purposely and self-excluded from these kinds of microfinance interventions. It may be time to rethink programme targeting and encourage participation among younger women. Our analysis shows that different types of IPV relate differently to intervention engagement, reinforcing the message that all types of IPV should be explicitly discussed in these kinds of interventions. Our results also indicate that women who experience IPV are more likely to attend all the gender training sessions. This suggests that there is opportunity to focus on sexual partner violence often persists even with promising interventions (Abramsky et al. 2014) and should therefore be addressed explicitly with topics built upon across all training sessions. The potential role of group support in augmenting reductions in partner violence highlights the importance of fostering group cohesion in this and other group-based interventions. Our findings reinforce that microfinance combined with complementary training programs are crucial to reduce IPV, rather than microfinance alone programmes—that have limited evidence of positive effect on women’s empowerment (de Mel et al. 2009; Niner 2019.).

## Conclusion

These exploratory findings suggest that the IMAGE intervention is acceptable and continues to have a positive influence on women’s intimate relationships, in a scaled-up operational setting. In addition, the study provided further insights how IMAGE and similar interventions might be further strengthened or targeted for specific groups.

## Electronic Supplementary Material

ESM 1 (DOCX 69 kb)
