# Supplementary material for: Intervention with Microfinance for AIDS and Gender Equity (IMAGE): Women’s Engagement with the Scaled-up IMAGE Programme and Experience of Intimate Partner Violence in Rural South Africa
Source: Prev Sci. 2019 Dec 2;21(2):268–81. doi: 10.1007/s11121-019-01070-w (PMC6987051; doi:10.1007/s11121-019-01070-w)
Supplement: Supplementary file 1 — (DOCX 69 kb) [file 11121_2019_1070_MOESM1_ESM.docx]

| Table 1.Partner violence and abuse prevalence estimates, last 12 months and lifetime | | | | | | | | | | | | | | | | |
| --- | --- | --- | --- | --- | --- | --- | --- | --- | --- | --- | --- | --- | --- | --- | --- | --- |
|  | **n (%)** | **During the last 12 months** | | **Lifetime** | | **Last 12 months only** | |  |  |  |  |  |  |  |  |  |
|  |  | **Total** | | **n (%)** | **95% CI** | **n (%)** | **95% CI** |  |  |  |  |  |  |  |  |  |
|  |  | **n (%)** | **95% CI** |  |  |  |  |  |  |  |  |  |  |  |  |  |
| Number | | 860 | | 860 | | 860 | |  |  |  |  |  |  |  |  |  |
| Physical violence | 860 | 46 (5.4) | 3.9-7.1 | 128 (14.9) | 12.6-17.4 | 16 (1.9) | 1.1-3.0 |  |  |  |  |  |  |  |  |  |
| Sexual violence | 860 | 25 (2.9) | 1.9-4.3 | 65 (7.56) | 5.9-9.5 | 6 (0.7) | 0.3-1.5 |  |  |  |  |  |  |  |  |  |
| Physical and/or sexual violence | 860 | 57 (6.6) | 5.1-8.5 | 146 (16.98) | 14.5-19.7 | 18 (2.1) | 1.2-3.3 |  |  |  |  |  |  |  |  |  |
| Economic abuse | 860 | 77 (9.0) | 7.1-11.1 | 123 (14.30) | 12.0-16.8 | 15 (1.7) | 0.1-2.9 |  |  |  |  |  |  |  |  |  |
| Emotional abuse | 860 | 96 (11.2) | 9.1-13.5 | - | - | - | - |  |  |  |  |  |  |  |  |  |
| Controlling in last relationship | 860 | 448 (52.09) | 48.7-55.5 | - | - | - | - |  |  |  |  |  |  |  |  |  |


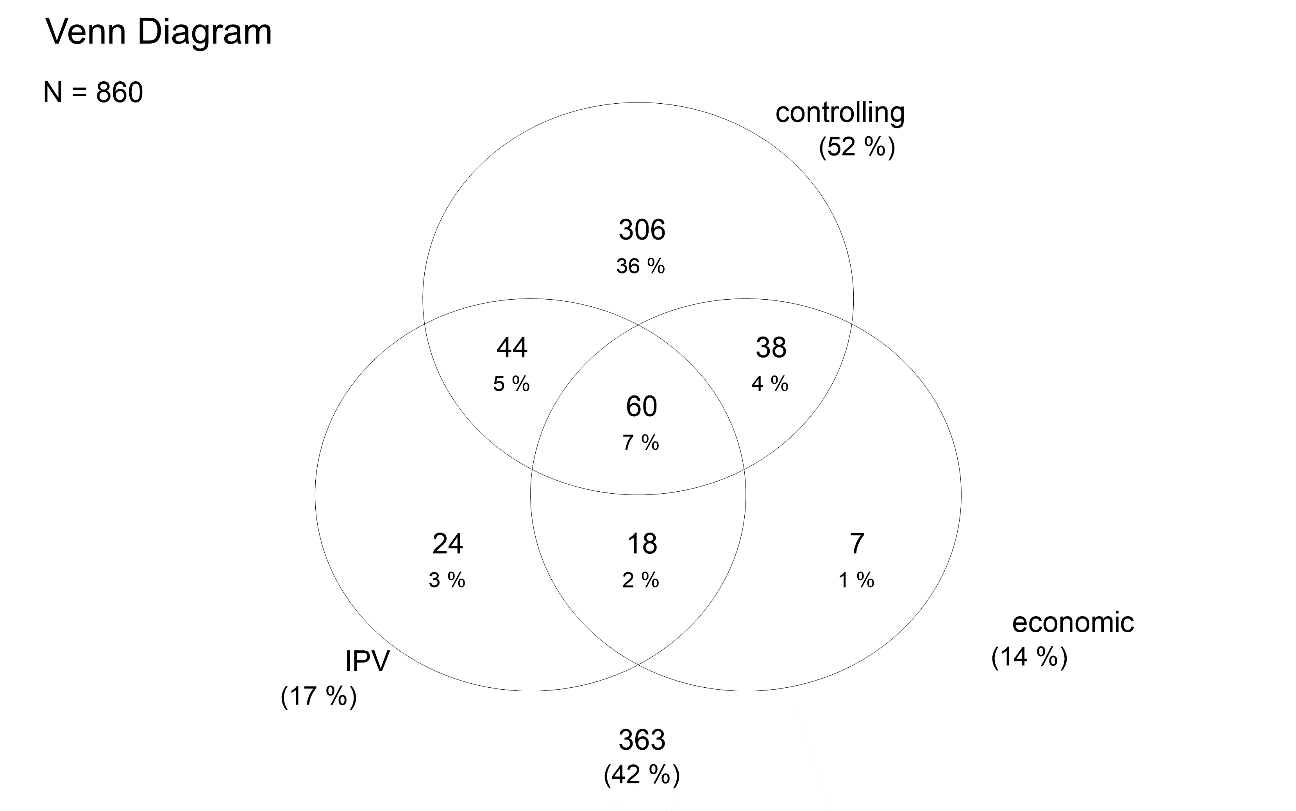


Figure 1: Venn diagram of overlap of types of lifetime partner physical and/or sexual violence (IPV) and economic abuse (economic), and partner controlling behaviour in last relationship (controlling), number and % of total cohort women.


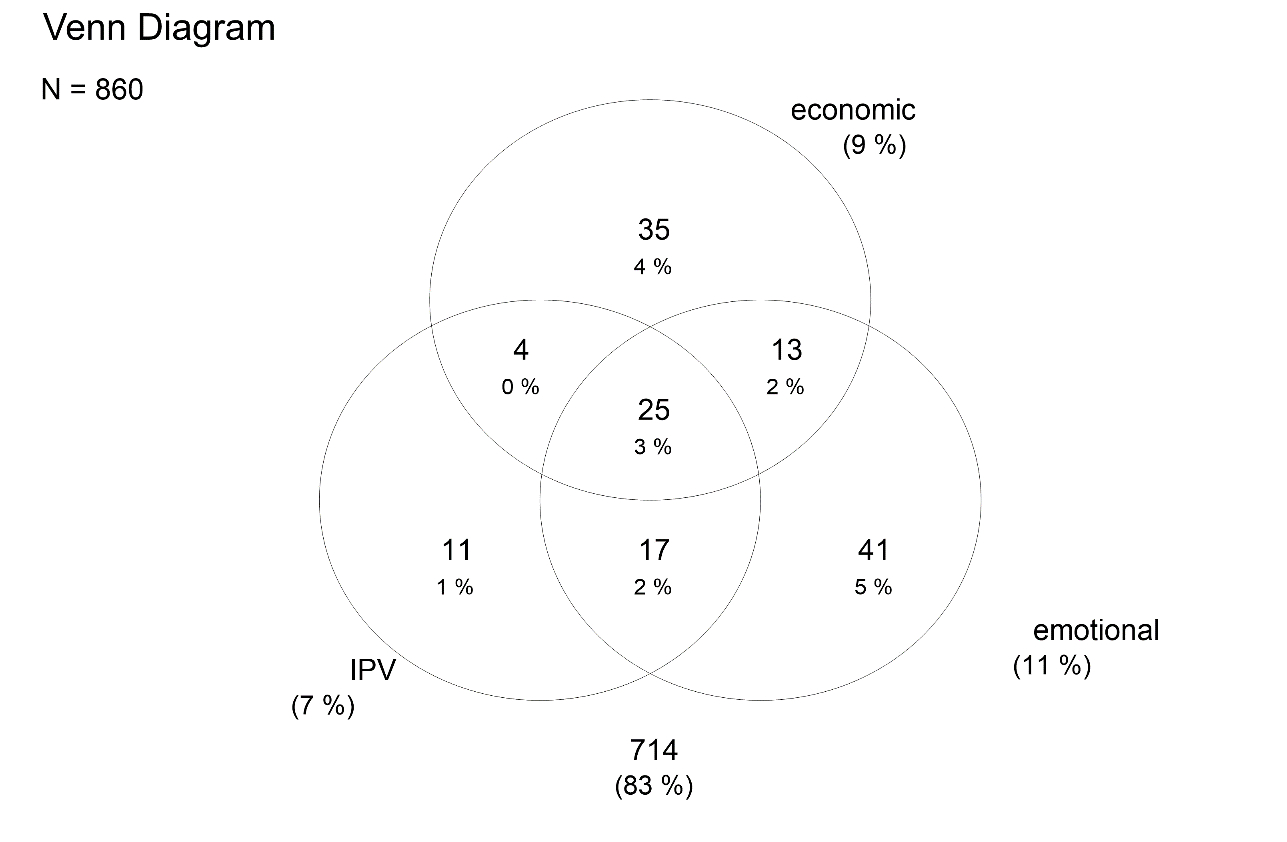


Figure 2: Venn diagram of overlap of types of past year physical and/or sexual partner violence (IPV) and economic abuse (economic), and emotional abuse (emotional), number and % of total cohort women.
